# Pain control in newborn: pharmacological interventions

**DOI:** 10.1186/1824-7288-41-S1-A18

**Published:** 2015-09-24

**Authors:** Paola Lago, Anna Pirelli, Daniele Merazzi, Elisabetta Garetti, Patrizia Savant Levet, Gina Ancora

**Affiliations:** 1 Woman's and Child's Health Department, AziendaOspedaliera-University of Padova, Padova, Italy; 2 MBBM Foundation, San Gerardo Hospital, Monza, Italy; 3 Woman's and Child's Health Department, Valduce Hospital, Como, Italy; 4 Woman's and Child's Health Department, Azienda Ospedaliero-Universitaria-Policlinico di Modena, Italy; 5 Mother's and Child's Health Department, Maria Vittoria Hospital, Torino, Italy; 6 Mother's and Child's Health Department, Infermi Hospital, AziendaOspedalieraRimini, Italy

## Background

Pharmacological interventions (PIs) are frequently used for pain control in newborn, particularly during respiratory assistance and in the postoperative period. However efficacy and safety of PIsare still not well demonstrated.

## Material and methods

To assess efficacy and safety of PIs for procedural pain in neonate, a literature search covering the period 2000-2015 via Medline and Cochrane Library database, was undertaken. PIs were evaluated in relation to intubation (INT), mechanical ventilation (MV)and postoperative analgesia (POp) in preterm and fullterm infants. Efficacy of PIs in controlling procedural pain and distress was assessed on validated pain scores as PIPP, DAN, CRIES, EDIN etc. Safety of PIs was evaluated in relation to reduced morbidity and on the Adverse Effects (AEs) reported. The authorsratedthe level of evidence (LOE) and strength of recommendations, according with GRADE system.

## Results

For tracheal intubation the efficacy of PIs in reducing stress and pain has been demonstrated for Remifentanil 2 mcg/Kg and Remifentanil 1 mcgr/Kg or Fentanyl 1-2 mcg/Kg plus midazolam 100 mcg/Kg. (N. 6 studies and N.344 newborns-LOE +++)[1,2]. In order to improve newborn's stability and reduce the time of intubation, Propofolcan be titrated at 1-2,5 mg/Kg in hemodinamically stable patient after the first 24 hours of life, or Fentanyl (F) administered at 2 mcg/Kg plus short half-life curare(N. 18 studies and N.1003 newborns-LOE ++/+++, *Recommendation* ↑↑) [3]. The use of opioids (F-or Morphine- M) in MV is effective in reducing the pain scores, however they may cause AEs as hypotension (M), prolonged ventilation, longertime to reach full enteral feeding (M&F) and adverse effects on neurodevelopmental outcome (M&F) in dose-dependent way; therefore they should be used selectively, when indicated by clinical judgment and evaluation of pain indicators (M 2 RCTs N. 1139 newborns, F 4 RCTs N.228 newborns- LOE+++, *Recommendation* ↑↑)[4,5]. In the postoperative period of major surgery, the use of opioids should be guaranteed at least in the first 48 hours (LOE++, *Recommendation* ↑); Tramadol does not appear to offer advantages over F regarding the efficacy, the duration of MV and the time to reach full enteral feeding. (LOE ++, *Recommendation* ↑).[6]Intravenous Paracetamolmay have an opioids-sparing effect and should be use in association with M or F.(LOE+++, *Recommendation* ↑↑))[7] (Table 1)

**Table 1:** Efficacy of Pharmacological Interventions in newborn

Pharmacological Interventions	Level of evidence		
	
	Grade of Recommendation		
**Opioids**	**INT**	**VAM**	**PostOP**

Fentanyl/ Fentanyl+ muscle relaxant	++	+++	+
	
	↑/↑↑	↑↑	↑

Remifentanil	+++	++	+
	
	↑	↓	↑

Morphine	++	+++	+
	
	↓	↑↑	↑

**Anesthetics**			

Ketamine	++	Non indication	Non indication
			
	↑		

Propofol*	++	Non indication	Non indication
			
	↑		

Tiopental*	++	Non indication	Non indication
			
	↑		

**Sedative**			

Midazolam in association with opioids**	++	+	+
	
	↑	↑	↓

Dexmedetomidine	Non indication	++	+
		
		↑	↑

**Other weak analgesic**			

Paracetamol**	Non indication	Non indication	+++
			
			↑↑

Tramadol	Non indication	Non indication	++
			
			↑

Legend.

INT =tracheal intubation, VAM = mechanical ventilation POp = postoperative pain

* Facilitating the procedure, reducing time required for INT

** Only in near term-term newborn

Level of Evidence (LOE): Very High ++++ (RCT), High +++ (RCT), Low ++ (Case series), Very Low + (Case report). Grade of Recommendation:strong or weak to use or strong or weak

## Conclusions

PIs are effective in relieving pain and stress from procedural pain in newborn but they use should be individualized and their effects monitored with validate pain scale to reduce the potential AEs.
